# Supplementary material for: Evidence supported by Mendelian randomization: impact on inflammatory factors in knee osteoarthritis
Source: Front Med (Lausanne). 2024 May 28;11:1382836. doi: 10.3389/fmed.2024.1382836 (PMC11165061; doi:10.3389/fmed.2024.1382836)
Supplement: Supplementary file 1 [file Data_Sheet_1.ZIP › data/GCST90274773_5e6/773.leaveoneout.pdf]

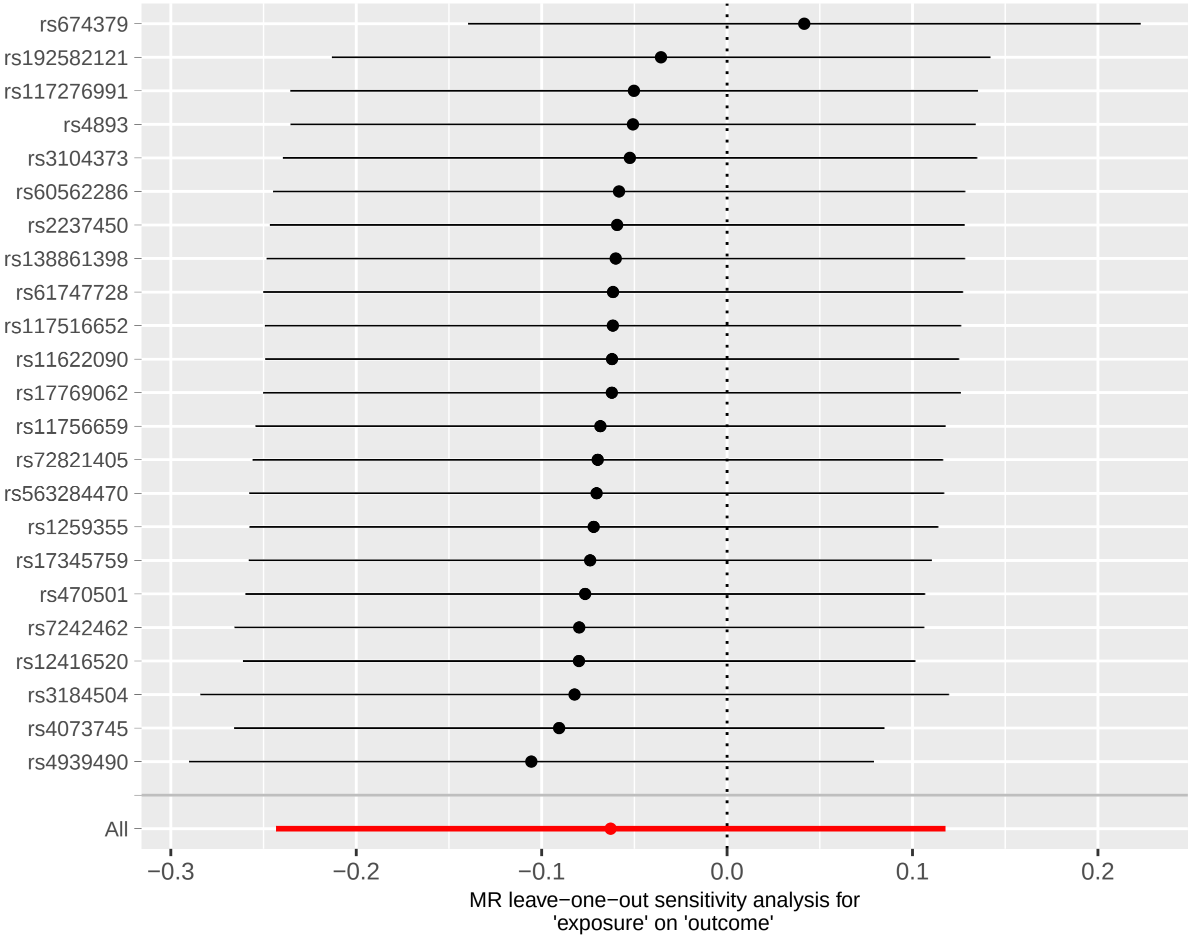
rs674379
rs192582121
rs117276991
rs4893
rs3104373
rs60562286
rs2237450
rs138861398
rs61747728
rs117516652
rs11622090
rs17769062
rs11756659
rs72821405
rs563284470
rs1259355
rs17345759
rs470501
rs7242462
rs12416520
rs3184504
rs4073745
rs4939490
All
−0.3
−0.2
−0.1
0.0
0.1
0.2
MR leave−one−out sensitivity analysis for
'exposure' on 'outcome'
